# Supplementary material for: Stacking Model‐Based Classifiers for Dealing With Multiple Sets of Noisy Labels
Source: Biom J. 2025 Mar 12;67(2):e70042. doi: 10.1002/bimj.70042 (PMC11898607; doi:10.1002/bimj.70042)
Supplement: Supplementary file 1 — Supporting Information [file BIMJ-67-e70042-s001.zip › density-based-ensemble-model-main/competitors_real_data/netlab-master/netlab-master/help/mlperr.htm]

Netlab Reference Manual mlperr


# mlperr

## Purpose

Evaluate error function for 2-layer network.

## Synopsis

```
e = mlperr(net, x, t)
```

## Description

`e = mlperr(net, x, t)` takes a network data structure `net` together
with a matrix `x` of input vectors and a matrix `t` of target
vectors, and evaluates the error function `e`. The choice of error
function corresponds to the output unit activation function. Each row
of `x` corresponds to one input vector and each row of `t`
corresponds to one target vector.

`[e, edata, eprior] = mlperr(net, x, t)` additionally returns the
data and prior components of the error, assuming a zero mean Gaussian
prior on the weights with inverse variance parameters `alpha` and
`beta` taken from the network data structure `net`.

## See Also

`mlp`, `mlppak`, `mlpunpak`, `mlpfwd`, `mlpbkp`, `mlpgrad`

---

**Pages:**
Index


---

Copyright (c) Ian T Nabney (1996-9)
